# Circulating miR-16 and miR-21 Levels in Multiple Myeloma: Prognostic Significance of Survival and Response to Lenalidomide Treatment

**DOI:** 10.3390/ijms25116065

**Published:** 2024-05-31

**Authors:** Annita-Ioanna Gkioka, Maria Tsota, Aspasia Koudouna, Alexandros Gkiokas, Christina-Aggeliki Mitropoulou, Aikaterini Palaiokrassa, Alexandros Alexandropoulos, Mavra Papadatou-Gigante, Vasiliki Bartzi, Thomais-Marina Tryfou, Petros P. Sfikakis, George V. Dedoussis, Marie-Christine Kyrtsonis

**Affiliations:** 1 Hematology Section, First Department of Propaedeutic Internal Medicine, Laikon Hospital, National and Kapodistrian University of Athens’ Medical School, 11527 Athens, Greece; anni.iwan.gk@gmail.com (A.-I.G.); aspakoud@hotmail.gr (A.K.); progoulantzaki@hotmail.com (A.G.); al.alexandropoulos@gmail.com (A.A.); mavra90@yahoo.com (M.P.-G.); vbartzi@yahoo.com (V.B.); thommais@hotmail.com (T.-M.T.); psfikakis@med.uoa.gr (P.P.S.); 2 Department of Nutrition and Dietetics, School of Health Science and Education, Harokopio University, 17676 Athens, Greece; mtsota75@gmail.com (M.T.); camitropoulou@outlook.com (C.-A.M.); katerina-060196@hotmail.com (A.P.)

**Keywords:** multiple myeloma, miR-16, miR-21, prognosis, lenalidomide, serum expression level, downregulation

## Abstract

MicroRNAs (miRNAs), particularly miR-16 and miR-21, play a crucial role in multiple myeloma (MM) pathogenesis by regulating gene expression. This study evaluated the prognostic significance of circulating miR-16 and miR-21 expression levels in 48 patients with MM at diagnosis treated with lenalidomide–dexamethasone (LD) compared with 15 healthy individuals (HI). All patients were treated with LD, 13 at first line and 35 at relapse, of whom 21 were tested twice at diagnosis and before LD initiation. The results revealed significantly lower levels of miR-16 and miR-21 in patients than in HIs, both at diagnosis and relapse, with decreased miR-16 levels at diagnosis, indicating improved overall survival (OS) (*p* value 0.024). Furthermore, miR-16 and miR-21 levels were associated with disease markers, while both correlated with the depth of response and mir-16 with sustained response to LD treatment. Ratios of both miR-16 and miR-21 expression levels (prior to LD treatment/diagnosis) below two predicted a shorter time to response (*p* = 0.027) and a longer time to next treatment (*p* = 0.042), respectively. These findings suggested a prognostic value for serum miR-16 and miR-21 levels in MM, as their expression levels correlated with disease variables and treatment outcomes.

## 1. Introduction

Multiple myeloma (MM) is a plasma cell malignancy characterized by monoclonal plasma cell proliferation in the bone marrow leading to significant morbidity owing to bone lesions in 80% of patients, anemia in 75%, renal failure in 20%, and hypercalcemia in 15% [1] (Rajkumar et al., 2016). Recent advances in MM treatment, characterized by the introduction of novel therapeutics, have markedly improved patient survival rates. These developments, along with innovations in cytogenetics and risk-stratification models, have progressively refined the prognostic assessment of MM [2] (Corre et al., 2021). Despite these advances, prognosis remains suboptimal for a subset of patients not adequately classified by current risk models, highlighting the need for novel prognostic indicators [3,4].

MicroRNAs (miRNAs) are a class of non-coding RNAs that regulate gene expression, predominantly post-transcriptionally influencing various cellular processes including proliferation, apoptosis, and cancer pathogenesis. Post-transcriptionally, miRNAs bind to untranslated regions of mRNA and regulate specific gene expression and act as proto-oncogenesor suppressor miRNAs [5,6]. Epigenetic processes such as DNA methylation, histone modification, and RNA modification influence the expression levels of miRNAs, while as a feedback function, miRNAs influence epigenetic enzymes such as DNA methyltransferases, histone deacetylases, and histone methyltransferases and operate as epigenetic regulators [7]. Although several studies indicate that miRNA expression patterns are related to the biology and clinical behavior of solid and hematologic malignancies (including multiple myeloma), the origin of miRNAs, the mechanisms of transit (secreted as free miRNAs actively or during cell apoptosis), and the most prominent role (intracellular or extracellular) are not well known [8]. Due to their stability as molecules, extracellular expression levels have been investigated for their potential prognostic impact in many tumors [9] (Chakrabortty A. et al.). However, limited research is available regarding the prognostic significance of circulating miRNAs in MM; miRNAs can be found in the serum in two forms, free miRNA and exosomal miRNA.

MiR-16 was initially associated with hematological neoplasms, such as chronic lymphocytic leukemia [10] and was among the first miRNAs examined in MM as part of Al Masri et al.’s study on miRNA expression analysis in MM [11,12]. A few studies in MM bone marrow have examined miR-16, showing downregulation and suggesting its potential as a prognostic marker [13].

Recent studies have shown that miR-21 is a proto-oncogene that plays a significant role in bone marrow-mediated growth, survival, and resistance of MM cells to therapy. Although, miR-21 has been studied in many cancers [14], data on serum levels in MM are extremely limited.

The role of miRNAs in MM represents an appealing approach for advancing our understanding of the molecular underpinnings of the disease and identifying novel prognostic tools.

Over more than a decade, the therapeutic landscape of MM has been revolutionized by the inclusion of lenalidomide in treatment regimens, initially in relapsed/refractory MM (RRMM), and then rapidly in both first-line and maintenance therapy strategies, which have been significantly improved. However, resistance to lenalidomide emerged as a challenge as these patients often have poor outcomes [15].

This backdrop sets the stage for our investigation of the prognostic relevance of miR-16 and miR-21 in newly diagnosed and relapsed MM, and their potential as biomarkers for lenalidomide treatment response. This study aimed to enlighten the prognostic landscape of MM and explore novel avenues for personalized therapy, potentially guiding more effective treatment strategies and improving patient outcomes. 

## 2. Results

### 2.1. Demographic, Clinical, and Laboratory Characteristics

In our study, we analyzed data at the time of diagnosis from 48 patients diagnosed with multiple myeloma according to the IMWG criteria. Among these patients, all were newly diagnosed, 13 received LD at the front line and 35 at relapse. Twenty-one patients were tested twice, at diagnosis and at relapse before LD administration. The patient cohort included 28 men and 20 women with a median age of 67 years (range, 40–85 years). All patients received a standard lenalidomide treatment protocol in combination with dexamethasone (LD). The distribution within the International Staging System (ISS) was relatively balanced, with ISS I accounting for 33%, ISS II for 28%, and ISS III for 39% of the total. The Ig type was IgG in 46%, IgA in 33%, and LC (light chain) in 17% of the patients, respectively. The majority of patients (80%) had at least one bone lytic lesion and 22% had hypercalcemia. The median number of treatment lines patients received was 3 (range, 1–6), median OS was 69.5 months (range, 2–188), median time to len response (TLR) was 3.5 months (range, 0–16), median time to next treatment after lenalidomide (TTNT after Len) was 14 months (range, 1–89). The analytic demographic, clinical, and laboratory characteristics of the patients are provided in Table 1 and Table 2.

### 2.2. MiRNA 16 and MiRNA 21 Results

**Figure 1:**
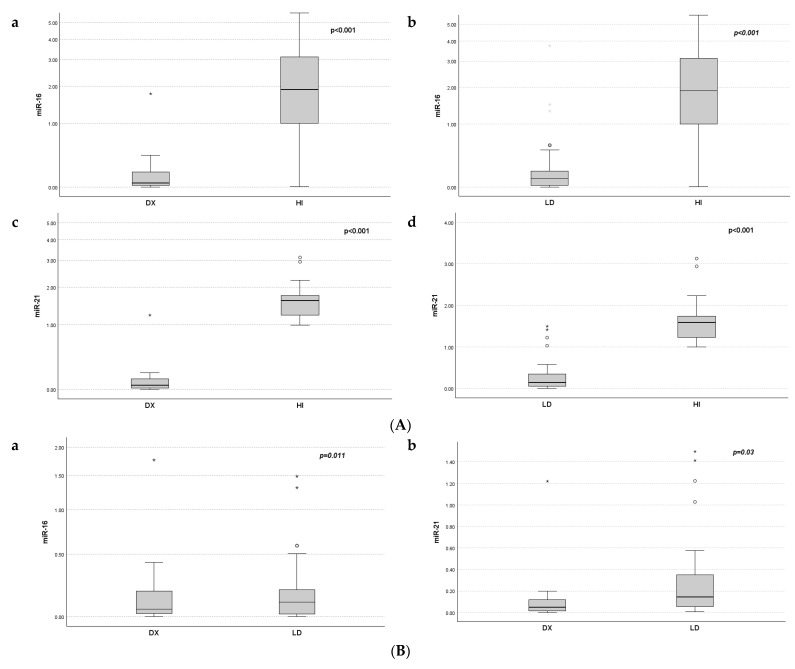
Distribution of miR-16 and miR-21 expression at diagnosis Dx, prior to LD treatment, and in healthy individuals (HI). (**A**): miR-16 (**a**,**b**) and miR-21 (**c**,**d**) expression differences between HI and MM patients at Dx and LD. (**B**): Differences in miR-16 (**a**) and miR-21 (**b**) expression between Dx and LD. * represents the higher values.

Serum expression of miR-16 and miR-21 was significantly decreased in MM patients at diagnosis compared to that in HI (miR-16 median 0.047 vs. 1.89, and miR-21 median 0.051 vs. 1.59, *p* < 0.001), respectively. Expression levels of miR-16 and 21 before LD initiation were also significantly downregulated compared to those in healthy individuals (miR-16 median 0.100 vs. 1.89, and miR-21 median 0.143 vs. 1.59, *p* < 0.001). The expression levels of miR-16 and miR-21 at diagnosis were significantly decreased compared to the levels at relapse (*p =* 0.011 and *p =* 0.03, respectively) (Figure 1B).

We used the median miR-16 and miR-21 expression levels at diagnosis to assess their association with clinical characteristics of MM. Significant correlations were observed between serum miR-16 expression levels and Cr (r = −0.473, *p =* 0.002), GFR (r = −0.466, *p =* 0.001), Cr ≥ 2 (r = −0.562, *p =* 0.002), hypercalcemia (r = −0.460, *p =* 0.003), B2M (r = −0.370, *p =* 0.021), and CRP (r = −0.356, *p =* 0.022). Moreover, serum miR-21 levels were significantly correlated with B2M ≤ 3.5 (r = 0.372, *p =* 0.017), Cr ≥ 2 (r = −0.301, *p =* 0.05), CRP (r = −0.330, *p =* 0.033), and neutrophil/lymphocyte ratio (r = −0.332, *p =* 0.045) (Table 3). Furthermore, lower expression of miR-16 was observed in patients with complex karyotype (three patients) and lower miR-21 expression in patients with del-17p (another three patients). Of course, the number of cases is too small to reach conclusions.

At the initiation of LD, serum miR-21 expression levels were significantly correlated with ISS (r = 0.468, *p =* 0.018) and treatment response (≥VgPR) (r = 0.453, *p =* 0.034). Additionally, there was a marginal correlation with Hb level (r = 0.377, *p =* 0.06). Conversely, serum miR-16 levels at LD initiation did not show any significant correlation with disease characteristics. However, expression levels that were twice the median value of miR-16 (×2) were associated with response to treatment (≥PR) (r = 0.427, *p =* 0.05) (Table 4).

To assess the impact of varying serum miRNA 16 and 21 levels at diagnosis (Dx) and LD initiation, the ratio of these two variables was calculated (miR-LD/Dx). The miR-16 LD/Dx ratio correlated with response to treatment (≥partial response) (r = 0.413, *p =* 0.05), and late relapse to LD beyond 24 months (r = 0.701, *p =* 0.0001). The miR-21 LD/Dx ratio was associated with biochemical relapse (r = 0.547, *p =* 0.015) and late relapse to LD beyond 24 months (r = 0.461, *p =* 0.035) (Table 4).

**Figure 2:**
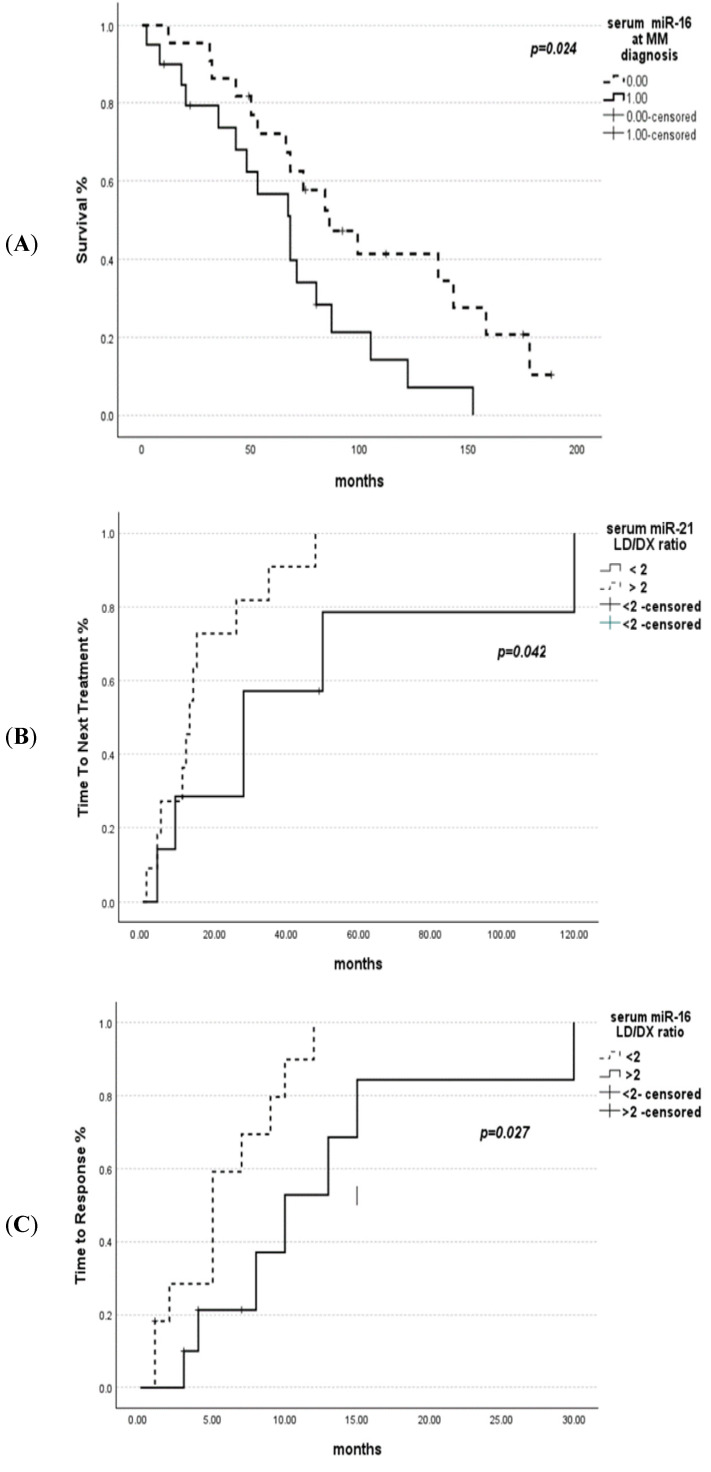
(**A**) Overall survival based on serum miR-16 levels at MM diagnosis. (**B**) Time to next treatment based on serum MiR-21 LD/Dx ratio: shorter TTNT for patients with a ratio >2. (**C**) Time to response based on serum MiR-16 LD/Dx ratio; shorter TTR for patients with a ratio <2.

Further analysis was performed to determine the impact of miR-16 and miR-21 expression levels at diagnosis on the prognosis of MM patients. miR-16 expression levels above median were associated with statistically improved OS (*p =* 0.024). However, miR-21 expression was not associated with overall survival (*p =* 0.346). At the initiation of LD treatment, a ratio of miR-16 LD/Dx below two was associated with a shorter time to response (TTR) (*p =* 0.027), and miR-21 LD/Dx below two with a longer time to next treatment (TTNT) (*p =* 0.042) (Figure 2). In order to seek weather miR levels could also predict response to other treatments, the same analysis was performed with the 35 patients that received a first-line treatment other than LD. Twenty-one patients received older conventional chemotherapy (17 VAD [vincristine, doxorubicin, dexamethasone], 4 MP [melphalan–prednisone]), and 14 patients received a VD (bortezomib–dexamethasone) regimen. No relationship was found between the expression levels of miR-16 and miR-21 at diagnosis and response to VAD or VD.

## 3. Discussion

Recent research has focused on miRNAs’ function and their involvement in epigenetic regulation of tumors. Studies have shown that miR-16 and miR-21 regulate cell cycle and are linked to the pathogenesis of MM.

Downregulation of miR-16 enhances cell proliferation through *AKT3* kinase (RAC-gamma serine/threonine-protein kinase), *MAP* (mitogen-activated protein) kinases, ribosomal protein S6, and *NF-κB* (nuclear factor-κB) activator MAP3KIP3 inhibition, and influences angiogenesis and apoptosis by increasing the expression of *VEGF* (vascular endothelial growth factor), *BCL2* (B cell Lymphoma 2) proteins, *Cyclin D1 (CCND1)*, *WNT3A* (Wnt family member 3A), and *MCL1* (induced myeloid leukemia cell differentiation) genes [13]. In MM, miRNAs-15a and -16 may disable disease progression by inhibiting various kinase pathways [4]. Moreover, miR-16 regulates the cell cycle and is involved in drug resistance in MM through induction of Interleukin-6 (IL-6) and cell cycle progression [16]. Recent research has shown that serum miR-16 expression levels differ amongst malignancies. miR-16 had lower differential expression levels in esophageal and stomach cancers compared to normal controls, indicating that miR-16 may operate as a tumor suppressor [17]. In MM, a few studies in bone marrow have demonstrated that miR-16 expression was downregulated compared with normal control cells and their expression levels had prognostic impact [18]. We studied serum levels of miR-16 which were significantly downregulated in newly diagnosed MM patients compared to normal controls (Table 4). Similar to our findings, circulating miR-16 was found to be downregulated both as free serum miR-16 [19,20], as well as in exosomal serum form [6] when compared to healthy individuals’ miR-16 expression.

miR-21 is involved in several signal transduction pathways, important to MM pathophysiology, such as the JAK/STAT-3 (Janus kinase signal transducer and activator of transcription) and NF-κB. Although circulating miR-21 has been shown to operate as a proto-oncogene in many cancers [12,21], the studies of serum miR-21 in MM are extremely limited with conflicting results [19,22] (Table 5). In one study, serum expression of miR-21 studied in 30 MM patients was significantly higher than in the monoclonal gammopathy of undetermined significance (MGUS) group (14 patients) and the control group [19]. On the contrary, the miR-21 in serum of MM, smoldering multiple myeloma (SMM), and MGUS patients was shown to be downregulated only in MM patients, indicating that considerable downregulation of this miRNA may be associated with subsequent events in disease progression. Furthermore, serum exosomes of miR-21 have been found downregulated in MM patients [23]. In our study, serum expression of miR-21 was significantly downregulated in newly diagnosed MM patients compared to normal controls (Table 6).

The expression levels of serum miRNA-16 and 21 measured right before LD treatment in the same patients remained significantly suppressed compared to healthy individuals, but a significant upregulation compared to the levels at diagnosis was observed. (Figure 1). Clinical characteristics, important in MM for the diagnosis and prognosis, were significantly correlated to serum miR-16 in our patients. This was the case for renal impairment, hypercalcemia, B2M, and CRP serum levels. The serum levels of B2M, renal impairment, CRP, and neu/lymph ratio were all associated with miR-21 at the time of diagnosis. miR-21 serum levels at relapse were associated with ISS stage. From previous studies, plasma levels of miRNA 16 were associated with B2M [24], ISS disease stage and PFS in myeloma [18]. In addition, serum miR-21 was significantly different according to the Durie and Salmon stages, correlating with B2M, IgG, and albumin levels [19]

Increased interest has been observed regarding the value of miRNAs in drug resistance in MM. In this context, the serum levels of distinct miRNAs were shown by Robak et al. [25] to be expressed differently in MM patients based on bortezomib sensitivity. Additional investigation has shown that miR-16 levels increased after melphalan and bortezomib treatment and decreased following the impact of IL-6 fluctuations, indicating their potential value as a response biomarker to therapies as well [26]. Likewise, increased expression of miR-21 in bone marrow stromal MM cells promoted resistance to dexamethasone, doxorubicin, or bortezomib through upregulation of NF-κB [27]. In contrast, the role of miRNAs in the modulation of the activity of IMiDs has been poorly investigated. Recently, argonaute 2 (AGO2), which plays a pivotal role in miRNAs’ maturation and function, was identified as a cereblon binding partner and it was found that the steady-state levels of AGO2 are regulated by cereblon [28]. This led to the concept that miRNAs had a synergistic impact with anti-MM factors and might be used to enhance anti-MM therapy or used as prognostic indicators. It is possible that the aforementioned mechanisms partly contributed to the increased miRNA expression found prior to LD treatment as compared to levels at diagnosis. In an attempt to determine the potential association of miRNA expression to lenalidomide, researchers showed that serum miRNA expression levels, including miR-26a-5p, miR-29c-3p, miR-30b-5p, miR-30c-5p, and miR-331-3p, were significantly downregulated in poor responders to treatment [29].

In addition, we observed that higher levels of miR-16 (twice the expression of median miR-16) correlated with response to treatment greater than PR and higher miR-21 levels with a response better than VgPR. Additionally, a higher ratio of expression levels before LD treatment/at diagnosis (LD/Dx) of both miR-16 and miR-21 showed significant association with late relapse to LD treatment (>24 months). Indeed, this is a very interesting finding taking into consideration that poor survival outcome has been linked to early relapse, irrespective of the depth of response to LD achieved [30].

Finally, our findings show that miR-16 and miR-21 levels have prognostic value for MM patients’ survival. Higher serum levels of miR-16 were associated with improved OS. Furthermore, a difference in serum levels between diagnosis and relapse MM was indicated by a lower than two miR-16 Dx/Rel ratio linked with shorter TTP and a miR-21 Dx/Rel ratio related with shorter TTNT, when evaluated prior to LD treatment. Lower expression of other miRNAs (miR-30b5p, miR-30c-5p, miR-193a-5p, and miR-331-3p) has been related to poorer overall survival or significantly shorter TTP after lenalidomide treatment [29].

One report by Rocci et al., 2014 [8] found that lower miR-16 levels are significantly related to shorter OS. Interestingly, when they compared intracellular miRNAs (from MM cells) and serum miRNAs from the same patients, no correlation was observed, thus suggesting a different origin for circulating miRNAs, such as normal immune/inflammatory or stromal cells. This may be the case also for the difference between low serum levels and intracellular increased expression levels of miR-21 in MM but this remains to be elucidated with further research. Such studies should include a much larger cohort of patients, be preferably prospective, and ideally standardize procedure for miRNA analysis in clinical samples.

## 4. Patients and Methods

### 4.1. Patients and Samples

In this study, we retrospectively enrolled a total of 48 MM patients at diagnosis and 35 at relapse prior to lenalidomide–dexamethasone (LD) treatment. All patients were treated in our department with LD, 13 at first line and 35 at relapse, of whom 21 were tested twice at diagnosis and before LD initiation. Systematic collection of serum samples was performed from both the patients and control group of healthy individu-als, ensuring that the samples were stored in a frozen state for subsequent analysis. Serum samples were collected at the time of diagnosis and before the first administra-tion of lenalidomide and dexamethasone. Our study’s inclusion criteria were based on the International Myeloma Working Group (IMWG) criteria for MM diagnosis, and all patients received a therapeutic protocol consisting of lenalidomide with dexame-thasone at either diagnosis or relapse. None of our patients received lenalidomide as maintenance therapy. The therapeutic protocol comprised lenalidomide (d1–d21) in combination with dexamethasone 20 or 40 md (d1, d8, d15, and d22) in a 28-day cycle (LD). The glomerular filtration rate (GFR) was determined using the Cockcroft-Gault equation.

### 4.2. MicroRNA Quantification

#### 4.2.1. RNA Isolation

Total RNA enriched for miRNAs was isolated from 100 μL of blood serum using the MagMAX™ mirVana™ Total RNA Isolation Kit (Thermo Fisher Scientific Inc., Waltham, MA, USA) ac-cording to the manufacturer’s protocol. Magnetic-bead technology was used to uni-formly recover high-quality RNA. The Implen P330 nanophotometer (Implen GmbH, Munich, German) was used to evaluate the concentration and purity of the extracted total RNA.

#### 4.2.2. cDNA Synthesis

A TaqMan® Advanced miRNA cDNA Synthesis Kit (Thermo Fisher Scientific Inc.) was used to generate cDNA, which uses universal primers to consistently amplify all targets, including low-expression miRNA targets, thereby increasing assay sensitivity.

#### 4.2.3. Quantification of Serum microRNA Expression

MiRNA quantification was performed for miR-16-5p and miR-21-5p. Advanced miRNA assays (for hsa-miR-16-5p (assay ID #477860_mir), for hsa-miR-21-5p (assay ID #477975_mir), and for cel-miR-39-3p (assay ID #478293_mir)) were used after cDNA synthesis to perform qRT-PCR with TaqMan® Fast Advanced Master Mix providing high specificity and the StepOnePlus™ Real-Time PCR System (Thermo Fisher Scientific Inc.). Each sample was analyzed in duplicate by qPCR. Expression-Suite™ Software version 1.3 was used for the analysis and calculation of relative gene expression using the comparative Ct (ΔΔCt) method and normalization of sample-to-sample var-iation to an exogenous control. For accurate and precise quantification of circulating miRNA levels, the Caenorhabditis elegans miRNA Cel-miR-39-3p was used as an exog-enous control. Finally, the relative levels of miRNA in patient samples were compared to the lowest value of healthy individuals’ miRNA (due to exceptionally elevated val-ues of miRNA expression in HI compared to MM patients), which was used as a refer-ence sample, and the final results were presented as fold change in expression using the 2ΔΔCt formula.

### 4.3. Statistical Analysis

The Mann–Whitney test was used to analyze differential miRNA expression be-tween MM patients and HIs. We used the median of miRNA expression levels as a cutoff point in the correlation and survival analysis. The median was the optimal cut-off point as it dichotomizes the examined variables into two equal groups for more ac-curate analysis results.

To assess the impact of varying serum miRNA 16 and 21 levels at diagnosis (Dx) and LD initiation, the ratio of these two variables was calculated (miR-LD/Dx). For the prognostic utility of this analysis, a ratio value greater than two was selected as the cutoff standard.

Clinical characteristics of the different groups and their associations with miR-NAs were evaluated using Spearman correlation analysis. The Kaplan–Meier method was used to calculate the overall survival (OS) curves according to miRNA-16 and miRNA-21 expression. Survival differences according to gene expression were ana-lyzed using the log-rank test. All statistical analyses were performed using SPSS software (version 28.0, IBM, Armonk, NY, USA). Statistical significance was set at *p* < 0.05.

### 4.4. Ethical Approval

The study was approved by the institutional review board (IRB) of our hospital (protocol number 191 and date of approval 21 February 2019). Informed consent was obtained from each patient prior to participation in the study, followed by a compre-hensive review of their medical records. Patient confidentiality was maintained throughout the study.

## 5. Conclusions

To summarize, we investigated the prognostic significance of circulating miR-16 and miR-21 levels in 48 patients at diagnosis with MM and in 35 at relapse time treated with LD. Twenty-one patients were tested twice at diagnosis and before LD initiation. Significantly lower levels of miR-16 and miR-21 were observed in patients compared to HI, both at diagnosis and relapse, with decreased miR-16 levels at diagnosis indicating improved overall survival (OS) (*p* = 0.024). The study also found that miR-16 and miR-21 levels were associated with markers of disease activity and response to LD treatment. The calculated expression ratio (prior LD treatment/diagnosis) below two for both miR-16 and miR-21 predicted a shorter time to response (*p* = 0.027) and a longer time to next treatment (*p* = 0.042), respectively. We report serum miR-16 and miR-21 levels in MM, which correlated with known disease prognostic factors and treatment outcomes. These findings are indeed preliminary, and further research is needed.

## Figures and Tables

**Table 1:** Patients’ characteristics at MM diagnosis.

Variable (n = 48)	Number (%)/Median (Range)
Age	67 (40–85)
Sex	
Male	28 (58%)
Female	20 (42%)
Ig Type	
IgG	22 (46%)
IgA	16 (33%)
Light Chain	8 (17%)
Biclonal	2 (4%)
Light Chain Restriction	
Kappa	31 (64%)
Lamda	17 (36%)
ISS	
I	15 (33%)
II	13 (28%)
III	18 (39%)

ISS: International Staging System.

**Table 2:** Main lab characteristics of MM patients at diagnosis.

Variable (n = 48)	Median (Range)
Hb g/dL	11.1 (6.7–15)
PLTs 10^9^/μL	212 (52–375)
Cr mg/dL	1 (0.5–4.6)
Hypercalcemia	10 (22%)
Alb	4.08 (2.5–5)
Bone Disease (determined by imaging)	36 (80%)
IgG mg/L	3360 (1850–8730)
IgA mg/L	3255 (944–6450)
IgM mg/L	17.3 (16.8–99.8)
FLCk mg/L	1398 (1.98–1510)
FLCλ mg/L	3.36 (0.08–2690)
FLCR	42 (0.9–52,750)
B2M mg/L	3.8 (2–16)
BMINF %	50 (10–95)
LDH UNL	3 (7%)
Neu 10^9^/μL	3455 (500–17,880)
Lymph 10^9^/μL	1610 (780–4340)
Neu/Lymph	2 (0.52–17.5)
CRP mg/L	3.45 (0.470–86.5)

Hb: Hemoglobulin, PLTs: Platelets, Cr: Creatinine, Alb: Albumin, Neu: Neutrophils, Lymph: Lymphocytes, FLCk: Free light chain—Kappa, FLCλ: Free light chain—Lamda, FLCR: Free light chain ratio, B2M: B2 microglobulin, BMINF: Bone marrow infiltration, LDH UNL: Lactate dehydrogenase—upper normal limit, Neu/Lymph: ratio of neutrophil to lymphocyte count, CRP: C-reactive protein.

**Table 3:** Correlation of serum miR-16 and miR-21 with disease characteristics of newly diagnosed MM patients.

Variables	miR-16 Dx *		miR-21 Dx *	
	R	*p*-Value	r	*p*-Value
B2M	−0.370	0.021	NS	NS
B2M < 3.5	NS	NS	−0.372	0.017
CRP	−0.356	0.022	−0.285	0.068
Neu/Lymph	−0.307	0.069	−0.332	0.045
Renal Failure	−0.410	0.008	−0.301	0.05
GFR	0.419	0.012	NS	NS
Hypercalcemia	−0.460	0.003	NS	NS

B2M: B2 microglobulin, CRP: C-reactive protein, Neu: Neutrophils, Lymph: Lymphocytes, GFR: Glomerular filtration rate, NS: Not Significant, (*) Dx: at diagnosis.

**Table 4:** Correlation between miR-16 and miR-21 before LD initiation and disease characteristics.

Variables	miR-16 LD ^v^		miR-21 LD ^v^		miR-16 LD ^v^/Dx *		miR-21 LD ^v^/Dx *	
	R	*p*-Value	r	*p*-Value	R	*p*-Value	r	*p*-Value
Biochemical Relapse	NS	NS	−0.371	0.068	NS	NS	0.547	0.015
ISS LD	NS	NS	0.468	0.018	NS	NS	NS	NS
Relapse > 24 months	NS	NS	NS	NS	0.709	0.0001	0.461	0.035
Response ≥ VGPR	NS	NS	0.453	0.034	NS	NS	NS	NS
Response ≥ PR	NS	NS	NS	NS	−0.413	0.05	NS	NS

ISS: International Staging System, VGPR: Very good partial response, PR: Partial response, (^v^) LD: Before LD initiation levels, NS: not significant, (*) Dx: at diagnosis.

**Table 5:** Serum miR-21 expression in MM.

Author	MM Patients (N)	HI	Method/Mirna Reference	MM Status	Drug	Relative Expression in NDMM	Regulation	PFS *p*	OS *p*
**Wang et al.** [16]	13 NDMM	12	qRT-PCR/nucleolar RNAs (RNU6, RNU38B, RNU44, RNU48, RNU66)	NDMM	NA	2.6	Downregulation	NA	NA
**Wang et al.** [19]	60 NDMM	30	qRT-PCR/NA	NDMM/ RRMM	MP VADTD	2.38	Upregulation in NDMM compared with HI/ Downregulation after therapy compared to NDMM	NA	NA

All studies tested sera samples and response to treatment was not available. Abbreviations: NDMM: Newly diagnosed multiple myeloma, PFS: Progression free survival, OS: Overall survival, qRT-PCR: Quantitative reverse transcription polymerase chain reaction, HI: Healthy individuals, RRMM: Relapsed/refractory multiple myeloma, MP: Melphalan–prednisolone, VAD: Vincristine–doxorubicin–dexamethasone, TD: Thalidomide–dexamethasone, NA: Not available.

**Table 6:** Prognostic significance of serum miR-16 expression in MM.

Author	MM Patients (N)	HI (N)	Method/miRNA Reference	Drug	Relative Expression in NDMM	Regulation	PFS *p*	OS *p*
**Rocci et al.** [6]	54 NDMM	67	qRT-PCR/hsa-miR-759 (5 pm)	VMP versus VMPT-VT	NA	Downregulation/cutoff point median value of HI expression	0.13	0.008
**Wang et al.** [16]	13 NDMM	12	qRT-PCR/nucleolar RNAs (RNU6, RNU38B, RNU44, RNU48, RNU66)	NA	3.1	Downregulation	NA	NA
**Li et al.** [15]	30 NDMM	10	qRT-PCR/cel-miR-39	Chemo	5.613	Upregulation/downregulation compared to NDMM (*p* = 0.492)	NA	NA
**Yyusnita et al.** [17]	14 NDMM/ 21 FU	7	qRT-PCR/miRNA, RNU6B	NA	2.084	Downregulation/no downregulation	NA	NA

All studies tested sera samples and response to treatment was not available. Abbreviations: NDMM: Newly diagnosed multiple myeloma, PFS: Progression free survival, OS: Overall survival, qRT-PCR: Quantitative real time polymerase chain reaction, HI: Healthy individuals, VMP: Bortezomib–melphalan–prednisolone, VMPT: Bortezomib–melphalan–prednisolone–thalidomide, NA: Not available.

## Data Availability

Data are contained within the article.
